# Supplementary material for: Disrupted cortex-wide dynamics impair motor planning in Shank3-mutant mice
Source: bioRxiv. 2026 Jan 14:2026.01.14.699439. Preprint. [Version 1] doi: 10.64898/2026.01.14.699439 (PMC12871110; doi:10.64898/2026.01.14.699439)
Supplement: 1 [file NIHPP2026.01.14.699439V1-supplement-1.pdf]

bioRxiv preprint doi: https://doi.org/10.64898/2026.01.14.699439; this version posted January 14, 2026. The copyright holder for this preprint (which was not certified by peer review) is the author/funder, who has granted bioRxiv a license to display the preprint in perpetuity. It is made available under aCC-BY 4.0 International license.

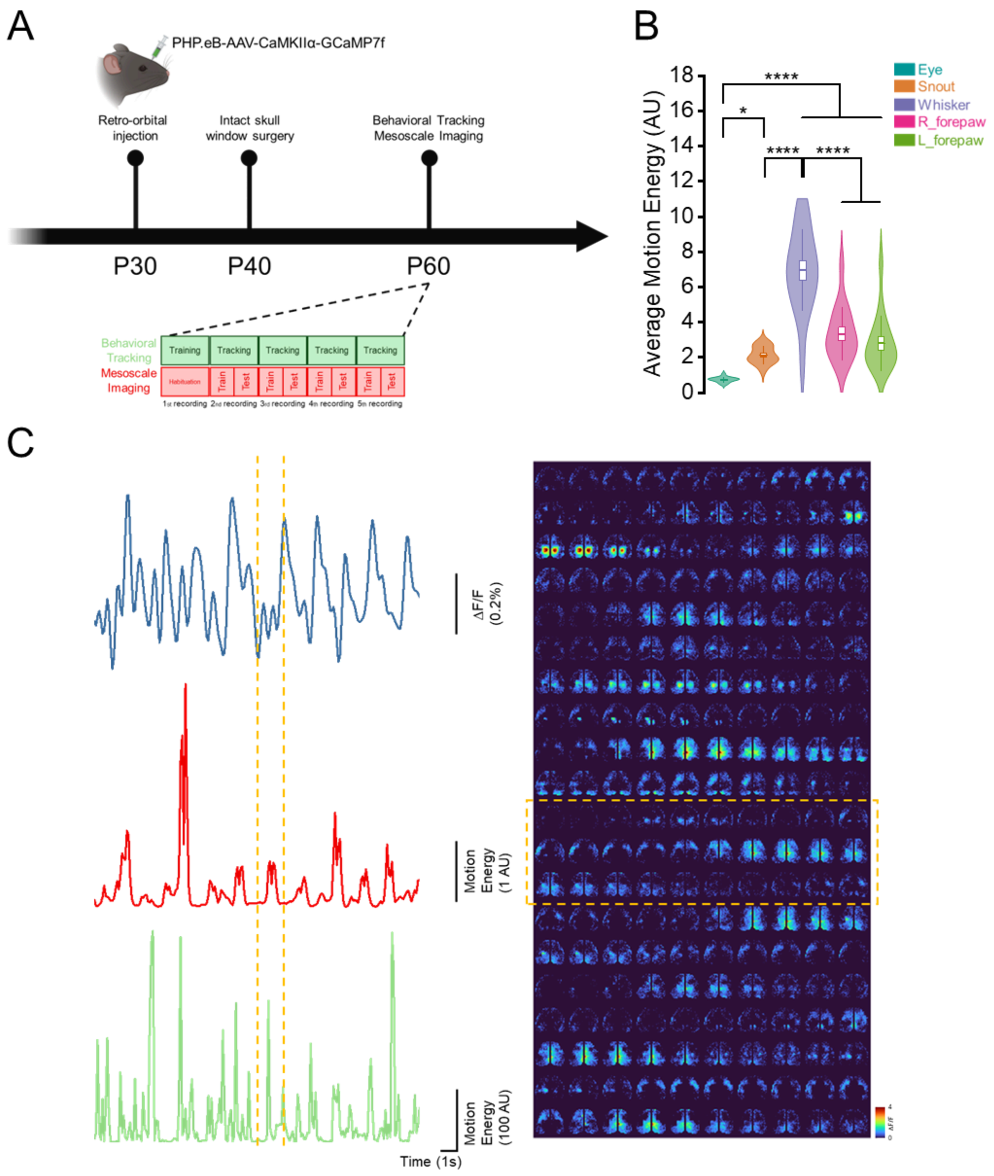

Supplementary Figure 1. Experimental timeline and motion energy calculation.
(A) Experimental timeline showing retro-orbital injection on PHP.eB-AAV at P30, followed by Intact skull window surgery at P40 and Imaging at P60. Five recordings were collected for each mouse. The first one was used to train DeepLabCut.
(B) Quantification of average motion energy for each ROI (eye snout, whisker, right forepaw, left forepaw). Asterisks denote significant differences.
(C) (Left) Example traces showing the comparison between ΔF/F (top), Cortex dynamics (middle) and Global Movements (bottom) through time. (Right) Example montage of cortical activity through time.

bioRxiv preprint doi: https://doi.org/10.64898/2026.01.14.699439; this version posted January 14, 2026. The copyright holder for this preprint (which was not certified by peer review) is the author/funder, who has granted bioRxiv a license to display the preprint in perpetuity. It is made available under aCC-BY 4.0 International license.

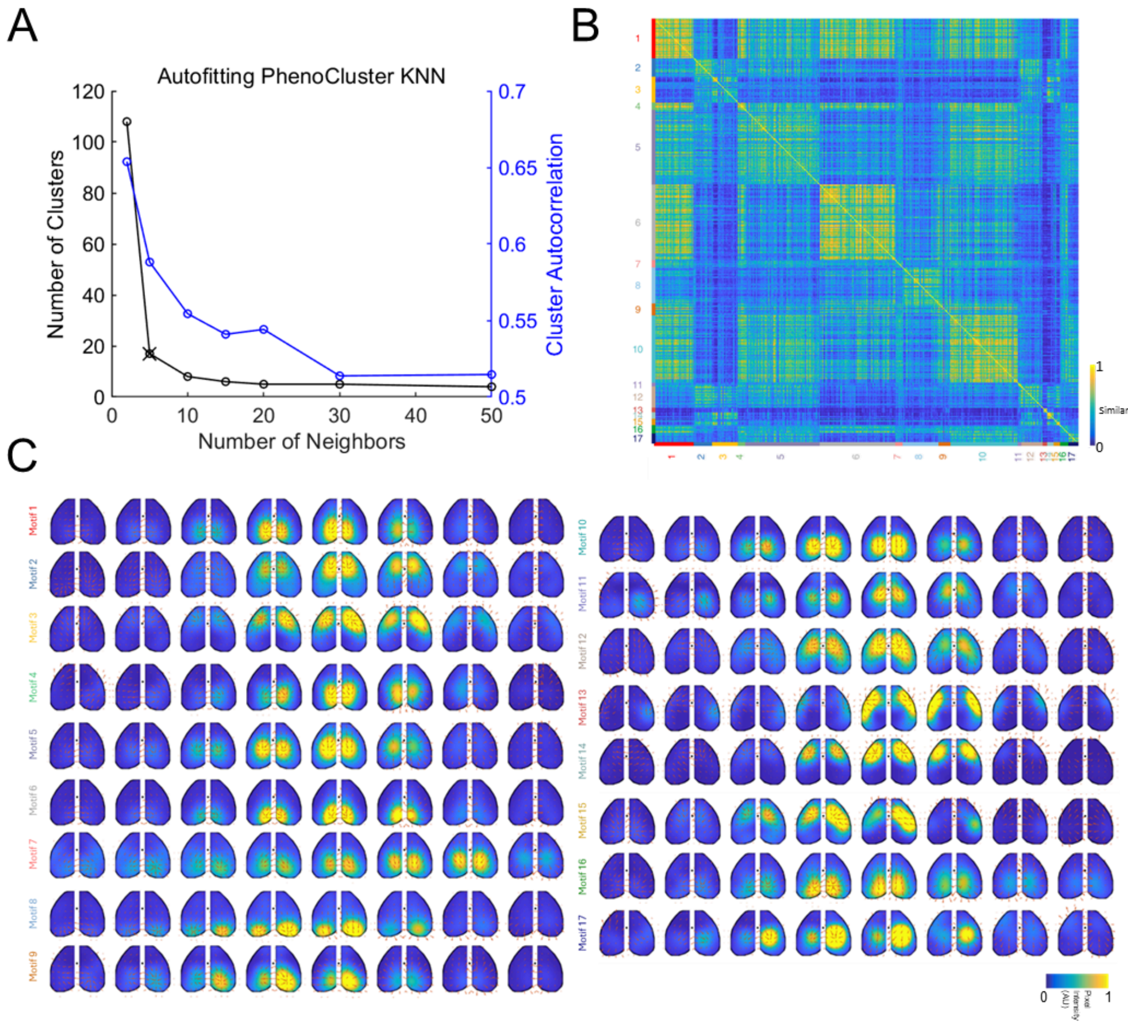

Supplementary Figure 2. Cluster parameters.
(A) Autofitting PhenoCluster KNN showing the optimal number of clusters for basis motifs (black cross, K=17).
(B) Pairwise correlation matrix showing the similarity across all motifs.
(C) Representation of all basis motifs extracted from discovered motifs.

bioRxiv preprint doi: https://doi.org/10.64898/2026.01.14.699439; this version posted January 14, 2026. The copyright holder for this preprint (which was not certified by peer review) is the author/funder, who has granted bioRxiv a license to display the preprint in perpetuity. It is made available under aCC-BY 4.0 International license.

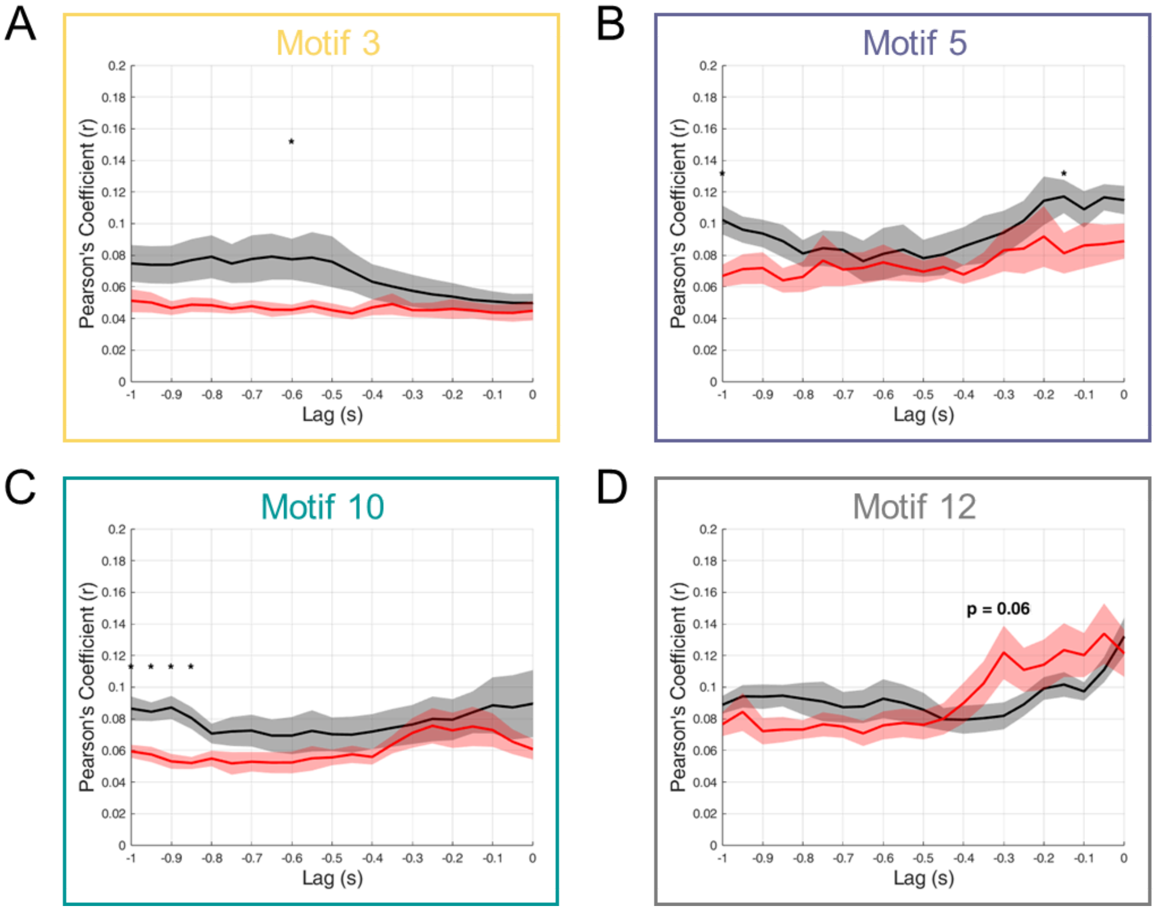

Supplementary Figure 3. Correlation between specific motifs dynamic and global movement.
(A-B) Correlation between global movement and cortical dynamics at negative lags for motif 3 (A) , motif5 (B), motif 10 (C) and motif 12 (D) in Shank3b+/+ (black) and Shank3b+/– (red) mice. Asterisks denote statistically significant differences.

bioRxiv preprint doi: https://doi.org/10.64898/2026.01.14.699439; this version posted January 14, 2026. The copyright holder for this preprint (which was not certified by peer review) is the author/funder, who has granted bioRxiv a license to display the preprint in perpetuity. It is made available under aCC-BY 4.0 International license.

	Motifs Discovery	Motifs Refit
K	28	17 (# basis motifs)
L	20	20
λ	0.0005	0
W	Random	Basis Motifs
H	Random	Random
λ_{Hortho}	1	1
λ_{Wortho}	0	0
Iterations	300	100
W_{fixed}	0	1
$W_{updated}$	1	0

Supplementary Table 1. CNMF parameters used for motif discovery and refit

bioRxiv preprint doi: https://doi.org/10.64898/2026.01.14.699439; this version posted January 14, 2026. The copyright holder for this preprint (which was not certified by peer review) is the author/funder, who has granted bioRxiv a license to display the preprint in perpetuity. It is made available under aCC-BY 4.0 International license.

	FC Shank3b+/+ (r)	SEM Shank3b+/+ (r)	P value	FC Shank3b+/- (r)	SEM Shank3b+/- (r)	P value
Baseline	0.615	0.013	//	0.659	0.011	//
Drop motif 1	0.596	0.013	0.001	0.637	0.011	0.0001
Drop motif 2	0.589	0.014	0.022	0.647	0.012	0.044
Drop motif 3	0.627	0.012	0.004	0.674	0.01	0.057
Drop motif 4	0.614	0.013	0.182	0.663	0.011	0.412
Drop motif 5	0.586	0.014	0.002	0.624	0.012	0.004
Drop motif 6	0.619	0.013	0.031	0.664	0.011	0.046
Drop motif 7	0.626	0.012	0.019	0.67	0.011	0.026
Drop motif 8	0.621	0.013	0.035	0.666	0.011	0.033
Drop motif 9	0.617	0.012	0.066	0.665	0.011	0.043
Drop motif 10	0.624	0.012	0.023	0.665	0.011	0.228
Drop motif 11	0.63	0.012	0.155	0.665	0.011	0.018
Drop motif 12	0.597	0.013	0.0001	0.638	0.012	0.0002
Drop motif 13	0.618	0.013	0.049	0.666	0.011	0.209
Drop motif 14	0.669	0.011	0.015	0.692	0.01	0.001
Drop motif 15	0.616	0.013	0.829	0.662	0.011	0.431
Drop motif 16	0.614	0.013	0.595	0.657	0.011	0.013
Drop motif 17	0.619	0.013	0.067	0.663	0.011	0.148

Supplementary Table 2. FC average values and SEM in Shank3b+/+ (black) and Shank3b+/- (red) mice. P values indicate significance when comparing each value with the value of the baseline for each group.
